# An open design for a low-cost open-loop subsonic wind tunnel for aerodynamic measurement and characterization

**DOI:** 10.1016/j.ohx.2022.e00352

**Published:** 2022-08-24

**Authors:** Ismail, Erlanda Augupta Pane, Reza Abdu Rahman

**Affiliations:** Department of Mechanical Engineering, Faculty of Engineering, Universitas Pancasila, Srengseng Sawah, Jagakarsa, DKI, Jakarta 12640, Indonesia

**Keywords:** Aerodynamic measurement, Open loop wind tunnel, Turbulence, Wind speed, Wind turbine

## Abstract

A wind tunnel is an essential device for aerodynamic modeling and measurement. High cost and relatively huge size with no open market design hinder the wind tunnel from being widely available in laboratory design for universities and small R&D companies, particularly in a developed country with limited research funding. Thus, most aerodynamic modeling and measurement are done by simulating through computer software which leads to high deviation as the nature of wind is unpredictable. This project aims to provide an open design for a relatively low-cost wind tunnel that universities and R&D companies can quickly adapt. An open design for an open-loop wind tunnel is presented in this article. The proposed wind tunnel design is specifically intended to help the researcher with the aerodynamic measurement with minimum cost for building, customizable design, and reliable measurement. The components, parts, and equipment use the widely available part, which can be obtained across the globe. Validation and characterization are done using software simulation and actual measurement through the device. The proposed design can meet the criteria for aerodynamic measurement and can help the researcher provide a better analysis by combining the actual measurement and software simulation.

## Graphical abstract

**Figure:**
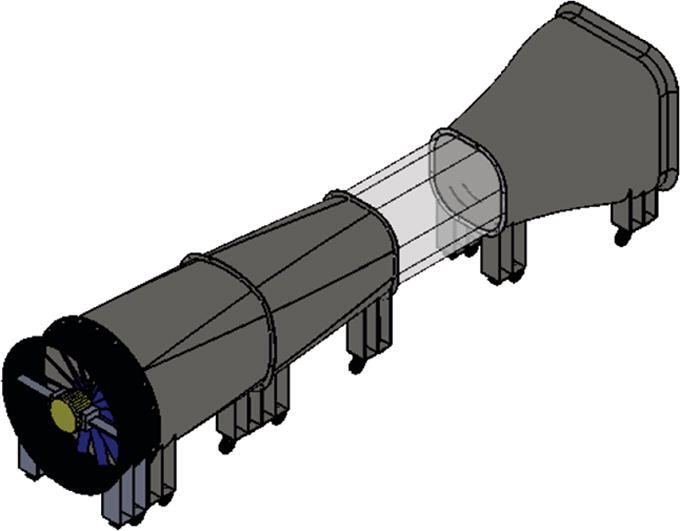

## Introduction

### **Specification Table**

**Table:** 

Hardware name	An open design for a low-cost open-loop subsonic wind tunnel for aerodynamic measurement and characterization
Subject area	• Mechanical engineering Educational tools as a laboratory equipment for aerodynamic measurement
Hardware type	• Measuring physical properties and in-lab sensors
Closest commercial analog	Open Circuit Wind Tunnel, *Close standard reference to:* NIST 1655 and ICAS-94-3.1.4
Open-source license	CERN OHL
Cost of hardware	USD $ 4,934
Source file repository	http://doi.org/10.17632/hdb93dwt2x.2

## Hardware in context

Global attention to wind energy utilization becomes a strong motivation for the researcher to improve the wind turbine design and optimize the turbine layout for a wind farm [1], including for small-scale wind turbine utilization for remote area [2]. The optimization for turbine design and wind farm layout can be done through numerical modeling using software and experimental methods using wind tunnels [3]. The numerical approach seems to be a favorite method since it is easier to conduct with minimum research cost [4]. Unfortunately, air as a moving fluid is hard to predict, so numerical modeling by using software varies significantly from the result of the prototype to the actual test [5]. Ideally, an experimental test of the turbine prototype or layout model must be tested in a wind tunnel to support the numerical modeling and minimize the deviation [6].

A wind tunnel as a media to evaluate the aerodynamic performance of a wind turbine has to meet the fundamental aspect of the aerodynamic test, which is suitable flow uniformity of the moving air inside the wind tunnel [7]. The characteristic of an open-loop wind tunnel can meet these basic criteria for an aerodynamic test with a wind tunnel where the overall turbulence intensity is less than 1 % and flow uniformity along the wind tunnel is 0.5–2 % [8]. The operating wind speed in an open-loop wind tunnel ranges from 1 to 10 m/s, highly recommended for testing the Vertical Axis Wind Turbine (VAWT). The main challenge with an open-loop wind tunnel as an aerodynamic instrument is costly with no open-source design or commercially available to rebuild the media. Furthermore, a typical wind tunnel operation is complex and requires a well-trained operator to operate it [9].

The above challenges can be solved by developing a new design for an open-loop wind tunnel that can be considered a low-cost design that can meet with the basic criteria for aerodynamic test and evaluation [10]. For the last decade, the research attention in aerodynamic and wind utilization increases significantly which is motivated for improving the aerodynamic design and harvesting wind energy through implementation small-scale wind turbine [11]. Small-scale wind tunnel is relatively easy in operation and maintenance, low turbulence and easy to remanufacture [12], [13], [14]. To support the development for small-scale wind tunnel, this work proposes an approachable design for an open-loop wind tunnel for aerodynamic evaluation, particularly for small-scale wind turbine, which can meet the fundamental requirement for an open-loop wind tunnel. The basic consideration for the design is recommendation according to National Institute of Standards and Technology 1655 [15] and ICAS-94-3.1.4 [16]. The design files, including step-by-step process and operation detail, are provided. The proposed design can easily manufacture without requiring specific materials and components. Thus, it can be easily replicated by the other researcher. The test section of the proposed design is acrylic, which can help observe the prototype during the testing. The test section is set as a knockdown model and can be easy to reassemble and help set the instrumentation and place the prototype in the test section.

## Hardware description

**Fig. 1:**
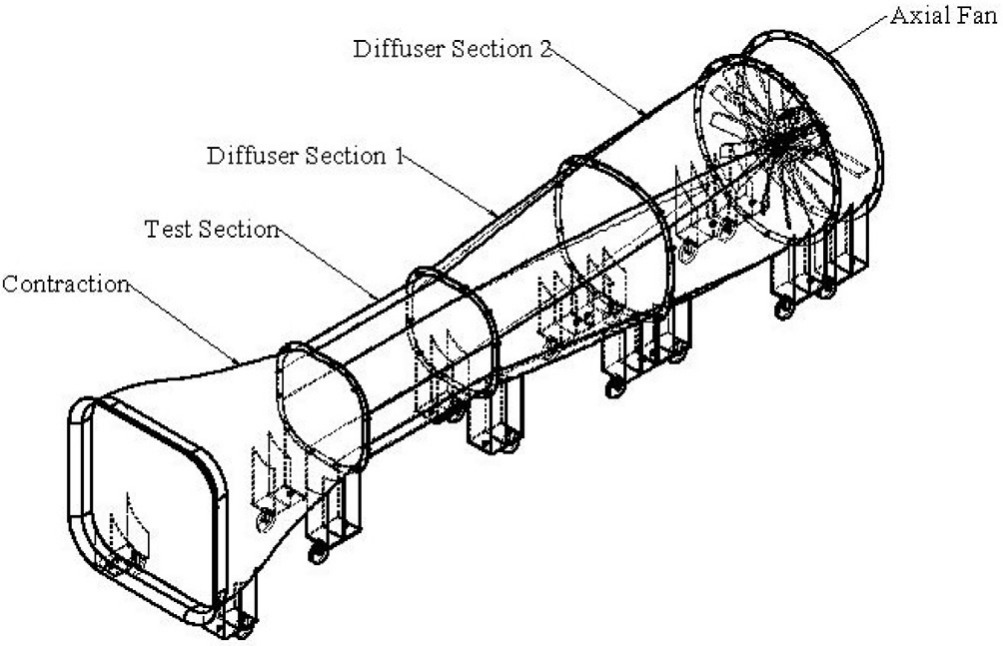
Main components of the proposed design wind tunnel.

The proposed open-loop wind tunnel design in this work is a suction–type wind tunnel for an aerodynamic test of a wind turbine with a working speed between 0 and 15 m/s. Fig. 1 presents the four major components of the designed wind tunnel in this work (diffuser sections 1 and 2 counted as one component).

High-quality aerodynamic measurement and characterization can be achieved by using the proposed model with the following advantages:

• Customizable honeycomb at the suction area provides a better fluid flow
• Easy to locate the instrumentation for flexible and direct measurement
• The spacious test section helps to accommodate large specimen
• Simple design, easy to manufacture with low turbulence intensity

### Contraction chamber

**Fig. 2:**
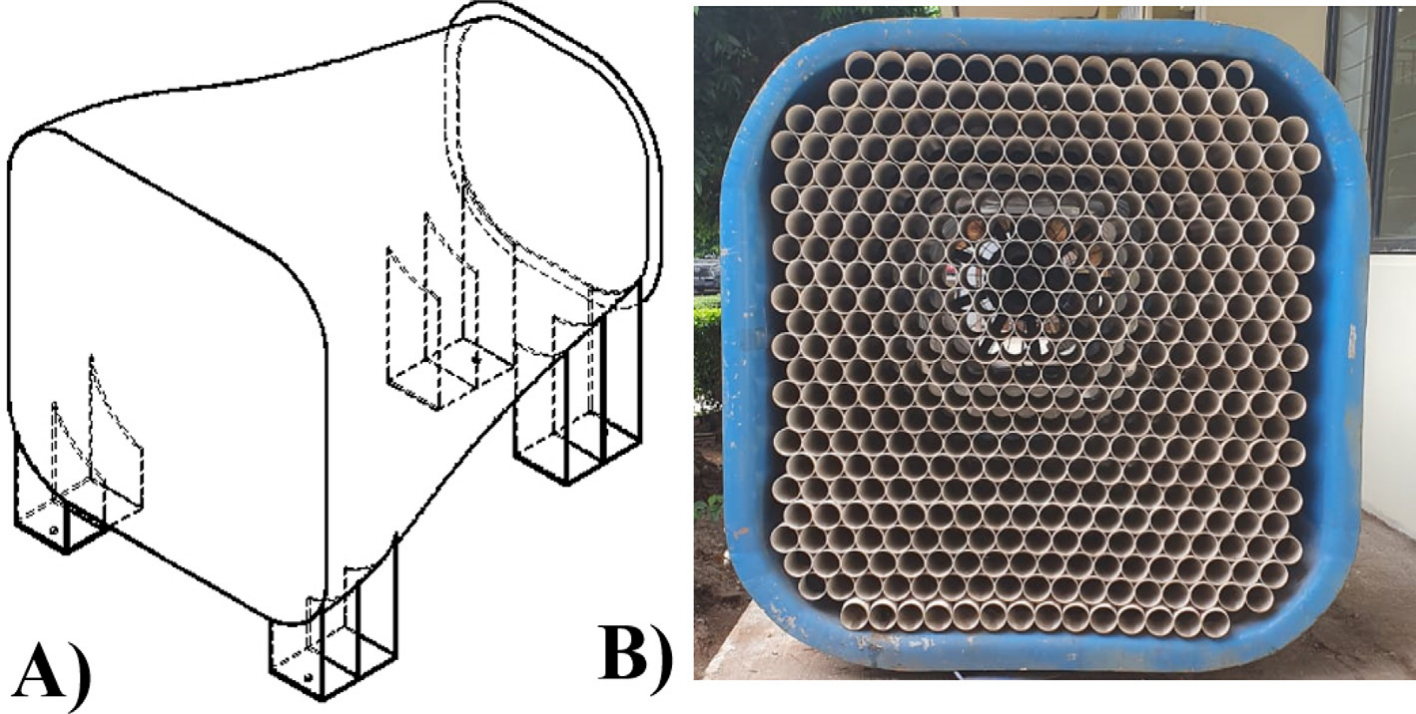
A) The contraction chamber of the proposed design, B) Example of honeycomb by using pipe arrangements.

The contraction chamber for a wind tunnel is designed to increase the air velocity entering the wind tunnel (Fig. 2**A**). The turbulence intensity tends to increase since the air enters the wind tunnel from the environment. A high turbulence intensity should be prevented; therefore, the contraction chamber is equipped with a settling chamber. In this design, the settling chamber can be set freely using a honeycomb made from wire mesh or a series of tubes. We recommend using a pipe with a diameter of ¾ − 1 in.. Again, the number and size of tubes, including mesh size (if using honeycomb), is entirely flexible and can be adjusted depending on the purpose. We provide an example for using pipe arrangements for the honeycomb (Fig. 2**B**).

### Test section

**Fig. 3:**
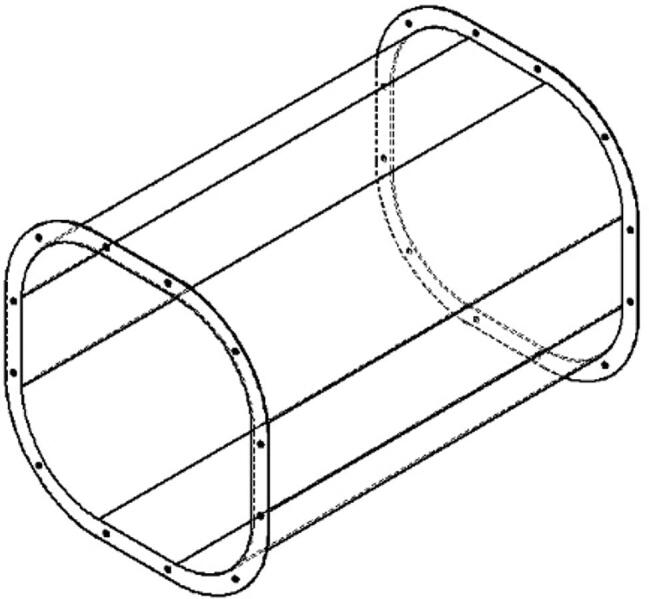
The test section of the proposed design.

The test section functions as the area to locate the prototype for the measurement (Fig. 3). The advantage of the proposed test section is the use of transparent acrylic. Using transparent acrylic comes with two main objectives: the researcher can observe the prototype directly during the measurement, and easy to reassemble to place the prototype before the test. The upper area of the test section can be easily opened/closed for placing the component, while the lower area can be combined with a nut/bolt for instrumentation and prototype holder. Using transparent acrylic also helps the researcher use a high-speed camera during the test. Furthermore, acrylic is relatively cheap and can be easily replaced when broken.

### Diffuser (1 and 2)

**Fig. 4:**
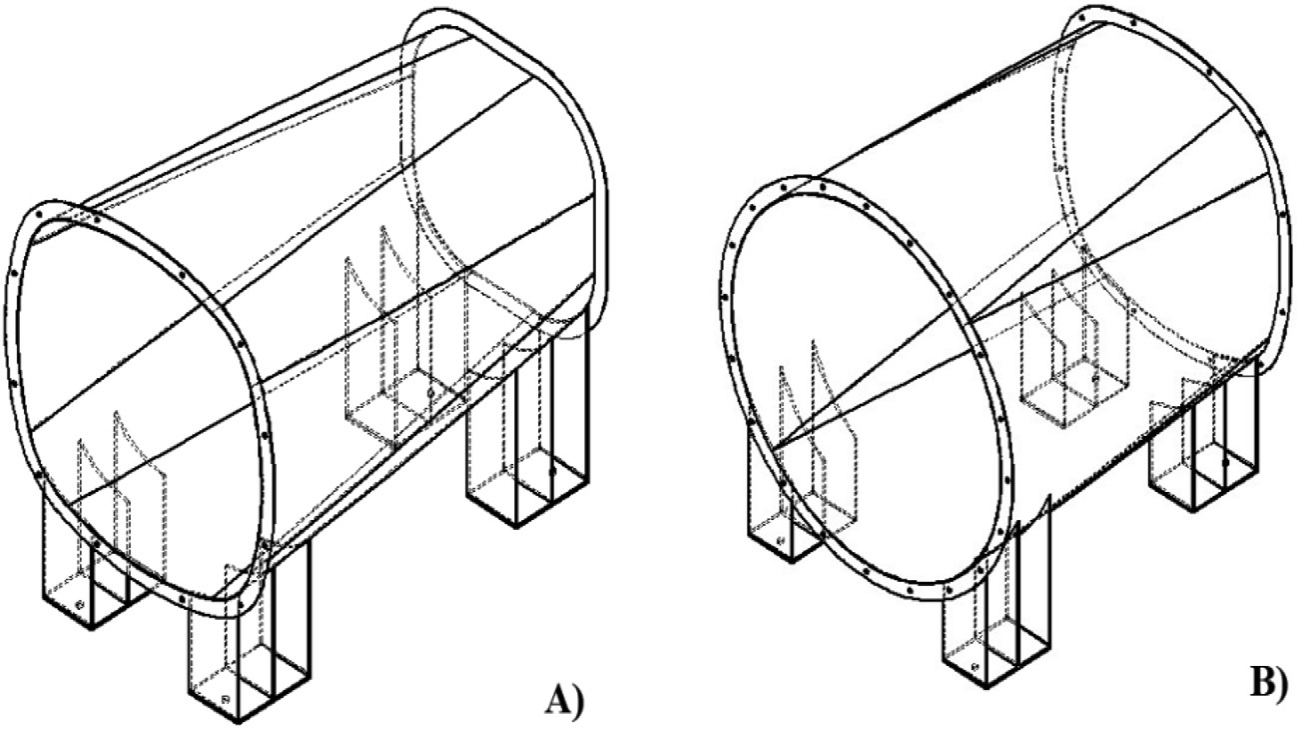
A) Model Diffuser Section 1, B) Model diffuser section 2.

The contraction chamber increases the air velocity that enters the wind tunnel, which is suitable for the tested prototype, but it is dangerous for the surrounding area of the wind tunnel if the wind is released directly to the outside area. The diffuser section is placed after the test section to minimize the effect of jet velocity from the wind tunnel (Fig. 4**A and B**). In principle, a diffuser is a device to reduce the wind velocity before exiting the wind tunnel. The designed diffuser is made into two areas to help the wind tunnel's manufacturing and assembly process.

### Axial fan

**Fig. 5:**
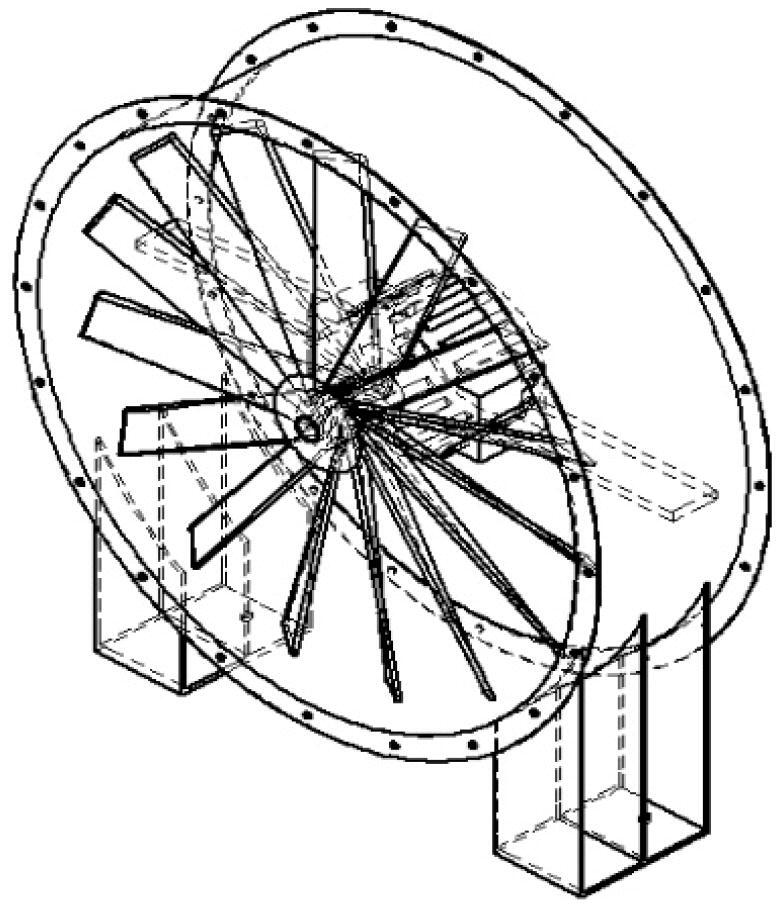
The axial fan in the designed wind tunnel.

The axial fan is located at the end of the assembly and is designed to provide sufficient wind speed for measurement (Fig. 5). The axial fan draws vacuum conditions within the wind tunnel to flow in the axial direction, parallel to the shaft about which blades rotate.

The rotation of the blade creates a pressure difference between the inside and outside area of the wind tunnel, making the air from the outside flow through the wind tunnel. The fan is set in suction mode, which means the air enters the wind tunnel from contraction chamber to the diffuser to minimize the turbulence intensity. A standard axial fan is used in this design which is widely available across the globe. They key point for choosing the axial fan is the ability to provide suitable wind speed within the tunnel by targeted speed 15 m/s (maximum).

## Design files summary

Table 1 presents the design files summary. There are two categories of the files design: 2D and 3D designs. The 3D files are provided as SLDPRT, STEP and STL while the 2D designs are provided as PDF for detail engineering drawing (DED). The 3D designs are intended to help the researcher to visualize the overall design of the model. The 2D designs are provided to give a detail engineering drawing for replication process.

**Table 1:** Design files summary.

Design file name	File type	Open-source license	Location of the file
Test_Section	3D: STL, SLDPRT, STEP 2D: pdf and JPG	CERN OHL	http://doi.org/10.17632/hdb93dwt2x.2
Fan			
Diffuser_1			
Diffuser_2			
Contraction			
Assembly_wind_tunnel			

## Bill of materials summary

The prices are based on currency rates Rp. 14,500/USD. Most of the materials can be easily found in the marketplace. We add sourcing materials for the fan controller, axial fan, anemometer and tachometer as reference (Table 2**.**). Furthermore, we don’t add relative items such as weld stick, glue, sandpaper for polishing, and polish material. We consider these items are depend on how the user build this design. For example, weld stick depends on the operator skill and type of weld. So, we believe this matter does not add significant value for the bills of material.

**Table 2:** Bills of Material.

Designator	Component	Number	Cost per unit	Total cost	Source of materials	Material Type	Ref.
Raw materials for contraction chamber, diffuser, ring and frame, adjustable feet	Mild steel (SS-400), thickness = 5 mm, 1220 mm × 2440 mm	6	$ 136.00	$ 816.00	Marketplace	Steel, SS-400	–
	Mild steel (SS-400), thickness = 2 mm, 1220 mm × 2440 mm	5	$ 120.00	$ 600.00	Marketplace	Steel, SS-400	–
	Mild steel (SS-400), thickness = 3 mm, 1220 mm × 2440 mm	2	$ 83.00	$ 166.00	Marketplace	Steel, SS-400	–
Test Section	Acrylic	1	$ 495.00	$ 495.00	Marketplace	Acrylic	–
	Connection lip (acrylic)	1	$ 130.00	$ 130.00	Marketplace	Acrylic	–
	Mold for test section	1	$ 70.00	$ 70.00	Marketplace	Wood	–
Paint	Base coat (kg)	12	$ 9.50	$ 114.00	Marketplace	Other	–
	Top coat (kg)	12	$ 11.40	$ 136.80	Marketplace	Other	–
Joint	Bolt for connection lip (M12)	36	$ 0.50	$ 18.00	Marketplace	Steel	–
	Bolt for axial fan (M14)	18	$ 0.62	$ 11.16	Marketplace	Steel	–
	Adjuster (M16)	16	$ 0.69	$ 11.04	Marketplace	Steel	–
Honeycomb	PVC pipe, 1.5 in.	4	$ 9.00	$ 36.00	Marketplace	PVC	–
Axial fan	5.5 kW, 960 RPM, 3 phase, belt drive	1	$ 1,300.00	$ 900.00	Ecommerce	Other	LINK
Fan controller	Non-linear control, voltage regulator, equipped with circuit breaker	1	$ 560.00	$ 560.00	Ecommerce	Other	LINK
Anemometer	Hotwire type	1	$ 260.00	$ 260.00	Marketplace	Other	LINK
Tachometer	Laser tachometer	1	$ 210.00	$ 210.00	Marketplace	Other	LINK

## Build instructions

The wind tunnel consists of four main components: contraction chamber, diffuser, test section, and axial fan. The axial fan is a ready-to-use component that can be easily attached to the diffuser section 2. Therefore, there are only three main components to be manufactured. The proposed design is prepared as a low-cost wind tunnel and easy to manufacture device, allowing the other researcher to manufacture the design easily. Thus, there is no special manufacturing process required. The overall process can be done using standard tools commonly available in workshops. The design files are prepared according to standard detail engineering design (DED). The building instructions can be followed according to the drawing. However, we highlighted the critical process for building the design.

1. The three components are manufactured separately
2. For the diffuser section 1, process the base material as the given dimension and weld (Fig. 6**A**), ensure the outer ring is fitted to the outside area of the diffuser case (Fig. 6**B**), weld the holder (Fig. 6**C**) to the case. The final assembly of the diffuser section 1 is similar to Fig. 6**D**.

**Fig. 6:**
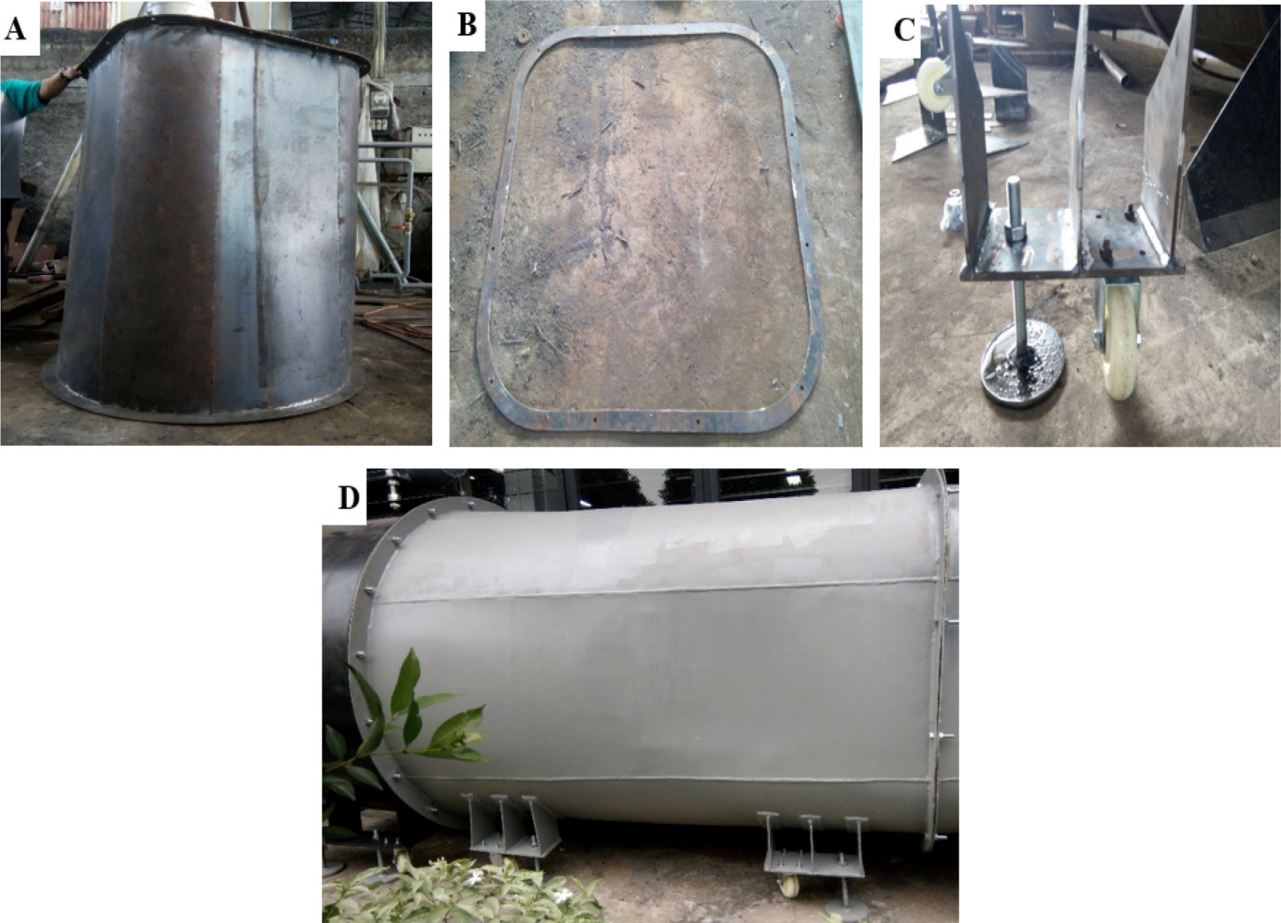
A) Diffuser section 1 case, B) Outer ring, C) Holder, D) Final assembly.
3. For the diffuser section 2, the same process is continued, weld the base material as the given dimension (Fig. 7**A**) and assemble with the outer ring and holder (Fig. 7**B**).

**Fig. 7:**
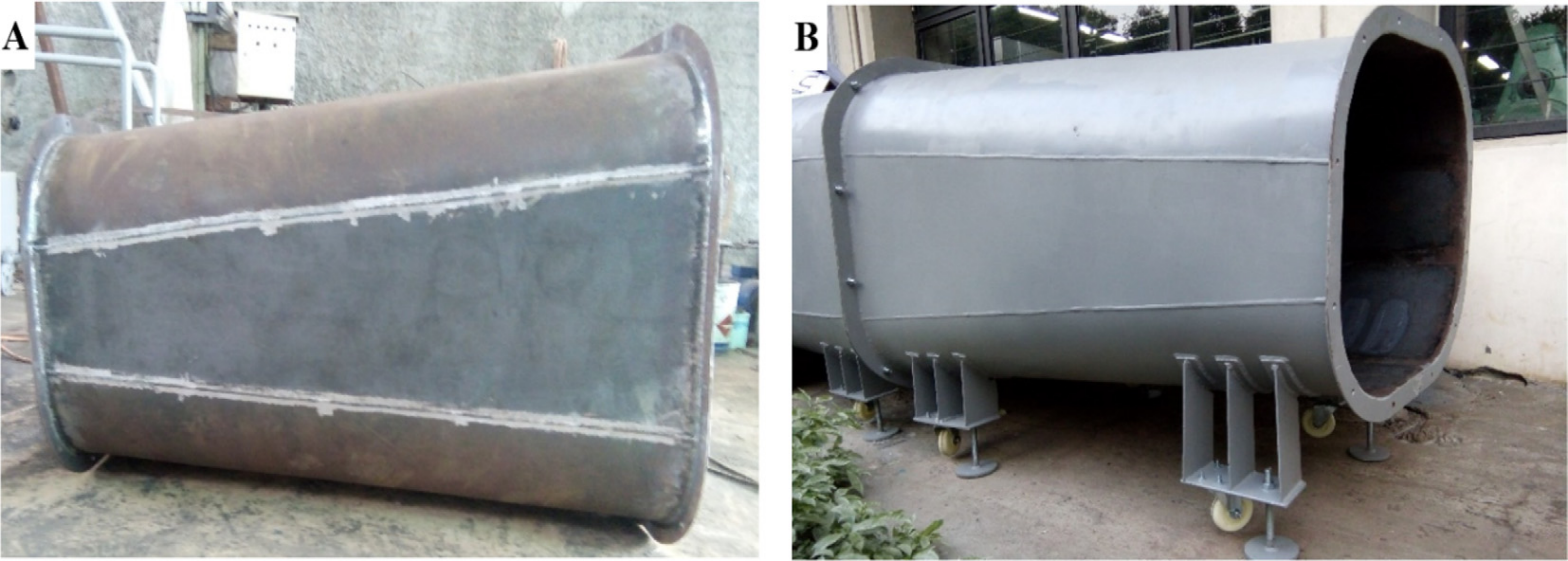
A) Diffuser section 2 (case), B) Final assembly.
4. The same process is continued for the contraction chamber, welding the base material as the given dimension (Fig. 8**A**) and assemble with the outer ring and holder (Fig. 8**B**).

**Fig. 8:**
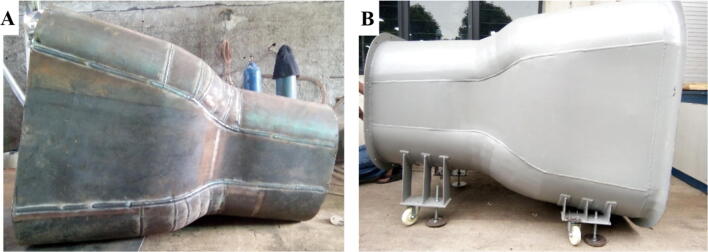
A) Contraction chamber, B) Final assembly.
5. The test section is made of transparent Polymethyl methacrylate (known as acrylic). First, build a mold for the test section according to the given dimension in the design file (Fig. 9**A**). Apply some heat for the acrylic and bend it to the mold, this process should be conducted carefully to ensure the final shape and dimension is appropriate. After bending the acrylic, check the final size (Fig. 9**B**). Once the final size meets the targeted size, join each end of the acrylic with acrylic glue and ensure it joint tightly. Create the connection lip (also made with acrylic) and join it with the test section (use acrylic glue). Attach the outer ring for the test section (Fig. 9**C**).

**Fig. 9:**
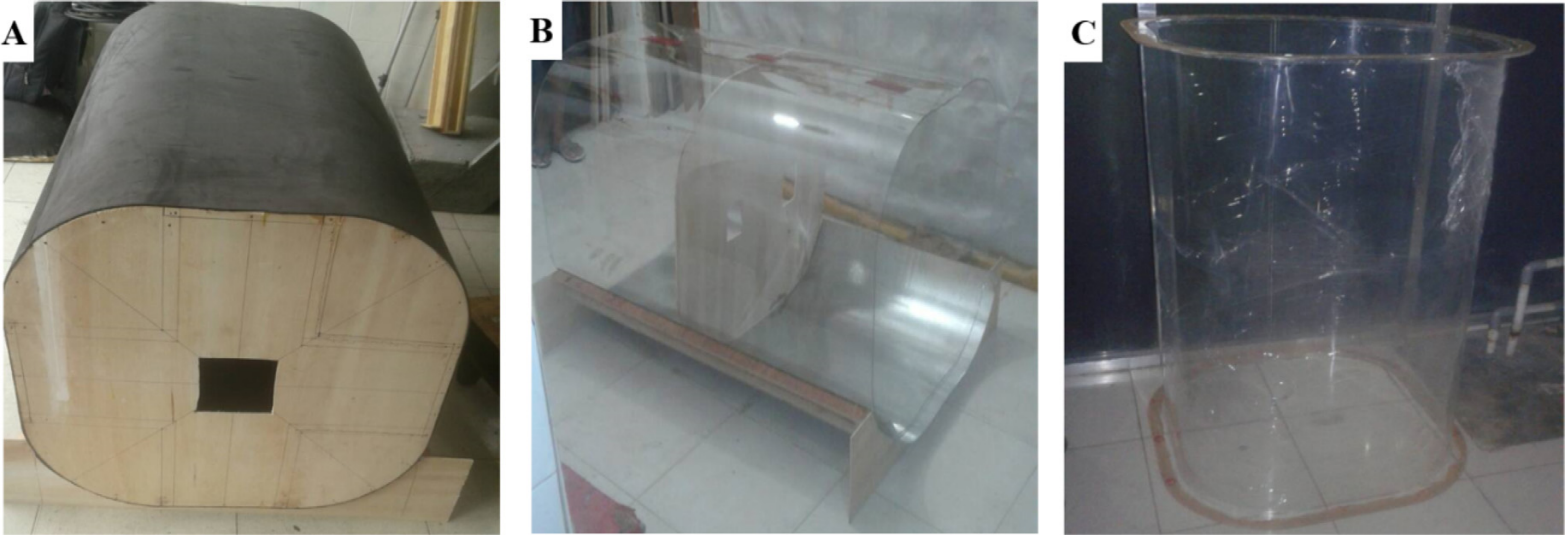
A) Molding for test section, B) Dimension check of the acrylic for test section, C) Final shape of the test section.
6. Ensure the inside area of each component is clean from any dirt that can occur from the process, sanding with abrasive material if necessary.
7. Apply base and top coat for the inside area of the diffuser and contraction chamber.
8. Assemble the all components, ensure each component has the same height, adjust the holder if necessary.
9. After finishing all processes, apply the external coating to protect the wind turbine from any dirt and rush (Fig. 10**.**).

**Fig. 10:**
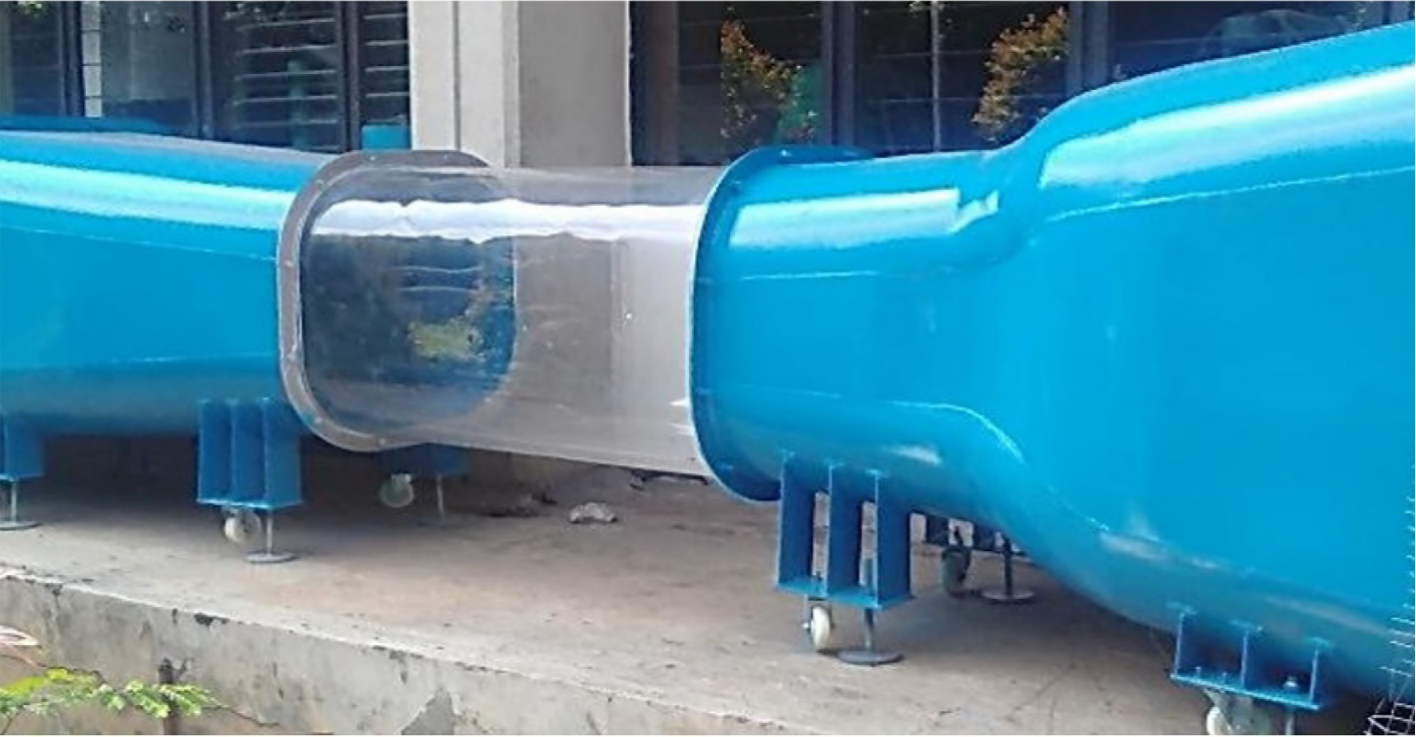
Final assembly for the open-loop subsonic wind tunnel.
10. Besides the mentioned step, several considerations should be conducted:
  a. Apply final polishing for the all-weld joint (for contraction chamber, diffuser section 1 and 2) that located in the inside area
  b. The polishing can be done by using sanding process and/or lapping process, the goal is made as smooth as possible for the joint area for the inside area of the wind tunnel
  c. If possible, try to conduct surface finish according to NISTIR 89-4088 [17]
  d. For painting, apply base coat, let it dry, check the surface quality. If necessary, repolish it, then apply top coat. Ensure the coated area is polished properly

## Operation instructions

### Panel controller

**Fig. 11:**
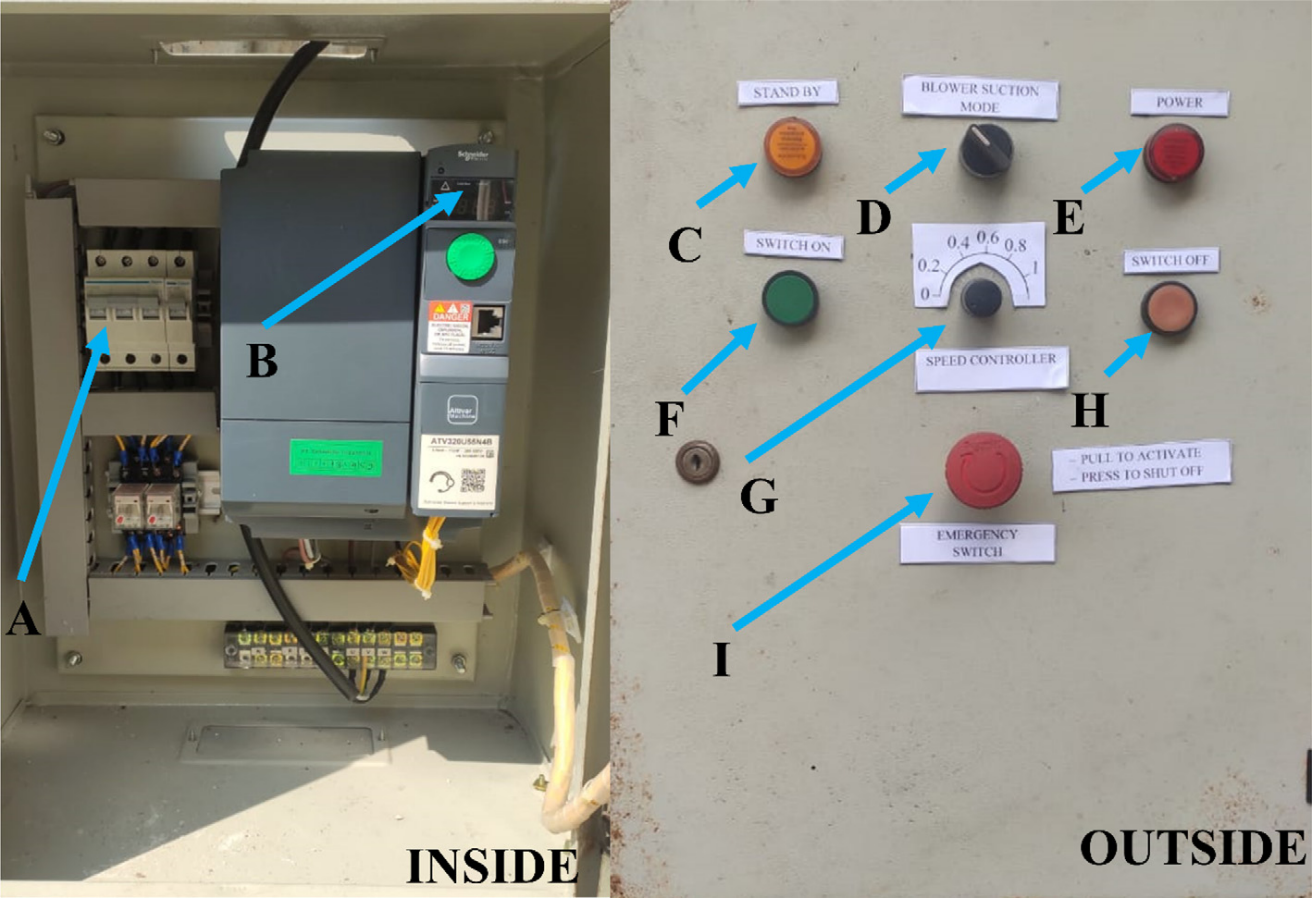
Box controller for controlling the axial fan.

Fig. 11 shows the box controller of the axial fan. The control panel for the fan is customizable 3-phase motor controller by using a non-linear voltage regulator. Inside the box, the controller is equipped with the main circuit breaker (A) and displays working voltage (B). The displayed working voltage is based on the knob controller (G). The outside area of the box controller is equipped with several switches, light indicators, and an emergency switch. The detailed function of the box controller is as follows:

1. The main circuit breaker (A) works as the main switch for the circuit (fan and its controller). When the main circuit board sets on, the light indicator (C) will illuminate to indicate that the system is in standby mode (engine standby).
2. Switch selector (D) is used for setting the operating mode of the axial fan, whether in suction (left) or blowing mode (right).
3. A knob controller (G) is used for controlling the fan speed by adjusting the working voltage of the fan. The working voltage is displayed in B. There are six different positions for the know controller: 0, 0.2, 0.4, 0.6, 0.8 and 1. The position is indicated the relative working voltage for the fan, where 0 indicates no voltage and 1 indicates the full speed of the working fan.
4. Light indicator (E) will illuminate when the emergency switch (I) is activated. It indicates that the fan is ready to work.
5. To turn on the fan, press switch (F) and to shut it off, press switch (H)
6. Safety measurements:
a Make sure the operating mode of the fan is set when the fan is not operating. Do not change the working mode of the fan when it is working since it will trigger the safety fuse to protect the circuit and fan from malfunction
b If the malfunction occurs during the process (Overspeed, knob controller does not work, another dangerous situation), push the emergency switch (I) to shut down the fan.

### Preparation

1. Open the outside cover on the contraction and axial fan side
2. Ensure the inside area of the wind tunnel is clean from any dirt
3. Ensure the settling chamber (tubes or wire mesh) is no blockage from dust and soil. Clean it if necessary
4. Set the MCB (main circuit breaker) on, ensure the light indicator (C) is illuminated, then adjust the operating mode of the fan by setting the switch controller (D) on the suction mode. Adjust the knob controller (G) at a position around 0.4–0.6. Pull the emergency switch (I), ensure the light indicator (C) is illuminated.
5. Press switch (F) to activate the fan. Let the fan works for about 5 min to clean the inside area of the wind tunnel from any dirt or soil. After 5 min, turn it off (Press switch H)

### Pre–Experiment

**Fig. 12:**
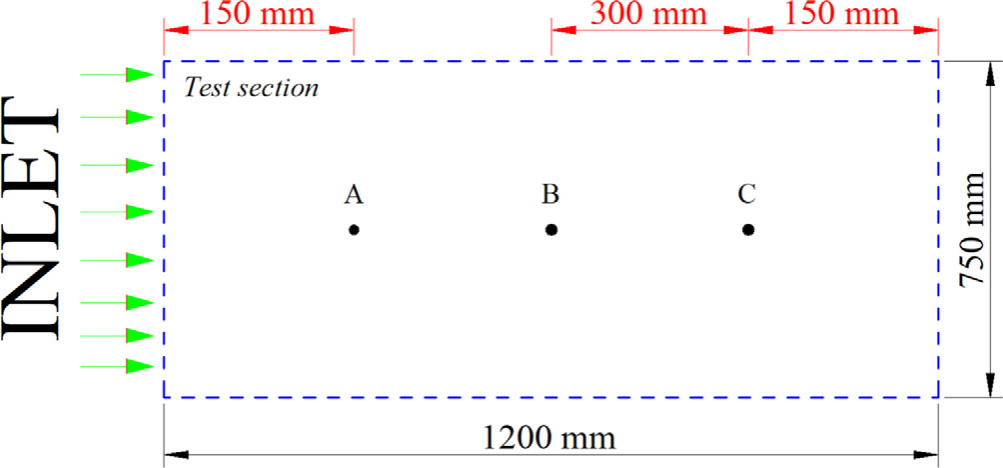
Schematic layout for placing the anemometer in the test section.

The fan speed is controlled by adjusting the working voltage of the fan. Thus, the wind speed inside the wind tunnel must be measured before experimenting. The measurement of the wind speed within the tunnel should be conducted carefully as the nature of the wind is hard to predict [18]. Wind speed measurement inside the test section should be measured in three different areas to ensure the reliability of the measurement (Fig. 12). Even the wind tunnel is equipped with a contraction and settling chamber to minimize the turbulence intensity. The wind speed measurement must be conducted more than once with a relatively small deviation. Please take into account that the position of the knob controller is not dedicated to determining the wind speed inside the wind tunnel. Thus, the measurement of the wind speed should be done as follows:

1. Define the targeted wind speed for the measurement
2. Sets the relative position of the knob controller, which is close to the targeted speed. Table 3 provides the relation between the position of the knob controller and the average wind speed based on the designed fan controller. For example, assume the targeted speed is 3 m/s, set the knob position at 0.2.

**Table 3:** Relative knob position and average wind speed based on the designed fan controller.

Relative Knob Position	Average wind speed (m/s)
0	0
0.2	2–4
0.4	5–7
0.6	8–10
0.8	11–12
1	13–16
3. For the measurement, put the anemometer at A (Fig. 12) in the test section and tighten the joint between the anemometer and the lower area of the test section to prevent leakage. Once it is done, turn the axial fan on and record the measured speed for 1 min. After the first measurement, turn the axial fan off, relocate the anemometer position from A to B and repeat the measurement (it is also applied for the C position).
4. Input the measurement result into a spreadsheet document to calculate the average wind speed in the test section are based on the knob controller position. The writing format may refer to Table 4.

**Table 4:** Spreadsheet format for calculating the average measured wind speed.

Target Speed (m/s)	3	
Knob Controller position	0.2	
Measurement Position	Measured Wind Speed	
	Min	Max
A	………	………
B	………	………
C	………	………
Average Wind Speed (m/s)	………	………

The ideal tolerance for the measurement is ± 0.2 m/s. For example, for the targeted speed of 3 m/s, the range of acceptable results of measured wind speed is 2.8–3.2 m/s. If the result is less or more than the acceptable range, readjust the knob controller and repeat the measurement.

### Experiment test with wind tunnel

Locate the prototype inside the test section area. The designed test section is spacious which make it possible to locate more than one prototype inside the test section area. Using more than one prototype is advantageous where it can be used for analyzing the turbulence effect of the prototype and layout configuration (specifically for windfarm optimization). Before starting the measurement, ensure the prototype is installed correctly in the test section, and there is no leakage from the joint and test section. The instrumentation for the measurement can be adjusted flexibly based on the test's purpose. For instance, for testing a wind turbine, a tachometer can be used to measure the turbine's rotational speed. A generator can be connected to the main shaft of the turbine from lower area of the test section to measure the power generation of the turbine. Artificial smoke (or fog) can be used to observe the fluid flow pattern inside the wind tunnel. The test should be done sequentially under a specific wind speed for the record.

## Validation and characterization

The critical aspect of a wind tunnel is the characteristic of pressure and velocity distribution in the test section. This aspect is crucial since it affects the turbulence intensity inside the test section area. If turbulence intensity in the test section area is excessive, it will affect the measurement accuracy for the prototype. Therefore, the validation and characterization of the designed wind tunnel are done in three different stages. The first stage is done by simulating the designed wind tunnel's turbulence intensity, pressure, and velocity distribution. The next step is experimental validation to ensure the feasibility of the designed wind tunnel, particularly for the test section area. The final stage is characterization using an actual wind turbine for various layout configurations under different wind speeds. The experiment results are compared to the simulation result to ensure the reliability of the result.

### Simulation for turbulence intensity, pressure and velocity distribution of the designed wind turbine

The first design stage is done by mathematical modeling to determine the dimension of each part of the wind tunnel. The critical part of the wind tunnel is the test section, as it is the area for placing the test specimen. The determination is intended to predict the flow characteristic, pressure and velocity distribution inside the wind tunnel. Mathematical modeling simulates the turbulence model using computational fluid dynamic (for example, Ansys Fluent 15.0 (licensed) or an opensource software like OpenFOAM®). Five different turbulence models were used to ensure the reliability of the simulation result: standard *k – ε* model, standard *k – ω* model, SST *k – ω* model, RSM dan LES model. The simulation found that the turbulence is relatively high, around 10 %. However, it is also found that the pressure and velocity distribution inside the test section is uniform, which makes the proposed design is suitable for aerodynamic measurement. The detail of the simulation process and the numerical equation can be found in this reference [19].

### Feasibility evaluation for test section area through experimental evaluation

The simulation through software shows the turbulence intensity is relatively high while, at the same time, it confirms a uniform pressure and velocity distribution inside the test section. Therefore, experimental validation is done to evaluate the feasibility of the test section under specific measurements. The measurement inside the test section is done repeatedly and carefully by using a specific reference to predict the turbulence intensity accurately. The experimental evaluation found that the average turbulence intensity inside the test section is 0.749 %. The experimental evaluation results confirm that the nature of turbulence is hard to predict and is not recommended to rely on simulation only and must consider the other parameter (i.e., pressure and velocity distribution). According to the experimental result, the test section is feasible for the aerodynamic test since the average turbulence intensity in the test section is less than 1 %. The detailed process and result for the feasibility evaluation through experiment test can be found in this reference [20].

### Experiment test for wind turbine

The actual measurement using a specific model wind turbine is conducted for validation on the designed wind tunnel. According to the rotor configuration inside the test section, there are three categories: single, inline, and staggered. The measurement uses a Vertical Axis Wind Turbine (VAWT) type Savonius rotor. The rotor design is set specifically by using the Myring Equation as a better approach for a modified Bach-type Savonius rotor [21]. The rotor speed from the actual experiment is compared by software simulation. The experiment is taken by following the procedure in Sections “*Pre–Experiment*” and “*Experiment Test with Wind Tunnel*”.

#### Single wind turbine

**Fig. 13:**
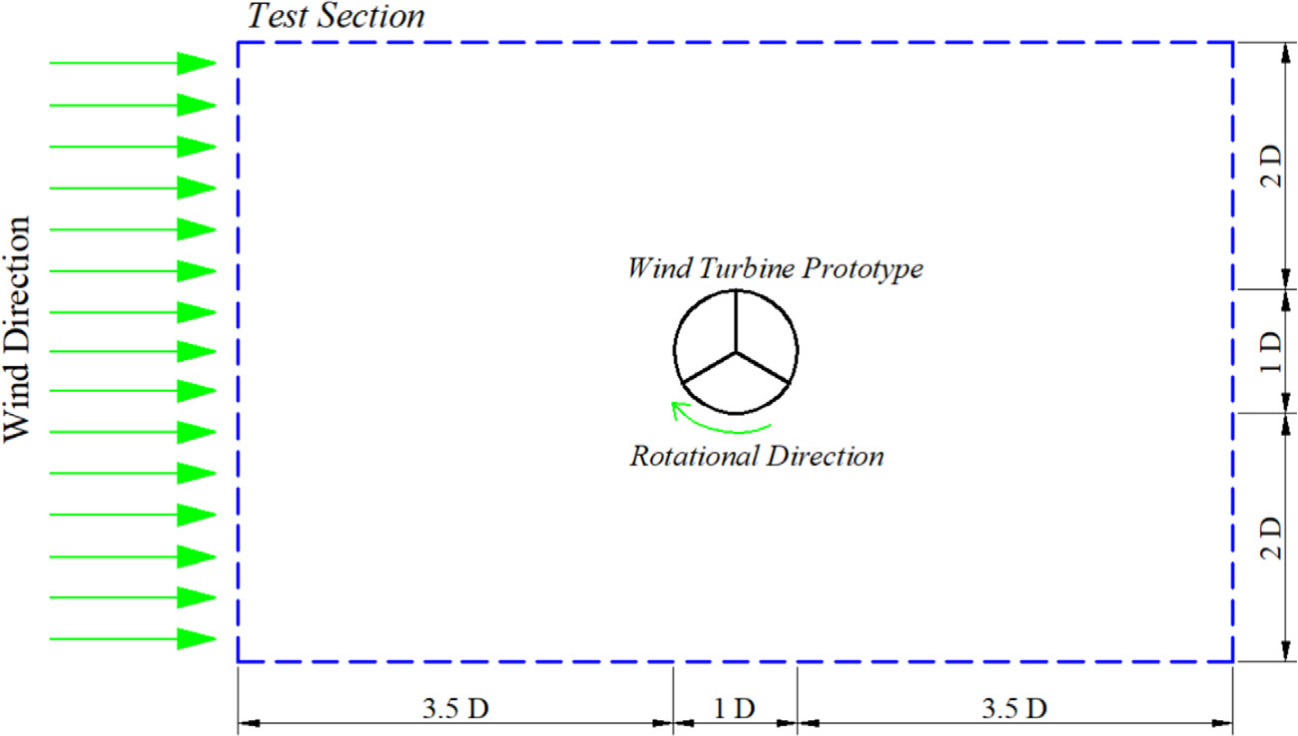
Layout for placing the specimen for single turbine measurement.

A single wind turbine is placed at the center point of the test section. The rotor's diameter (D = diameter = 15 mm) is taken as a reference for locating the specimen in the test section (Fig. 13). The exact parameter (wind speed, rotor position and dimension) from the actual measurement is set for software simulation.

**Fig. 14:**
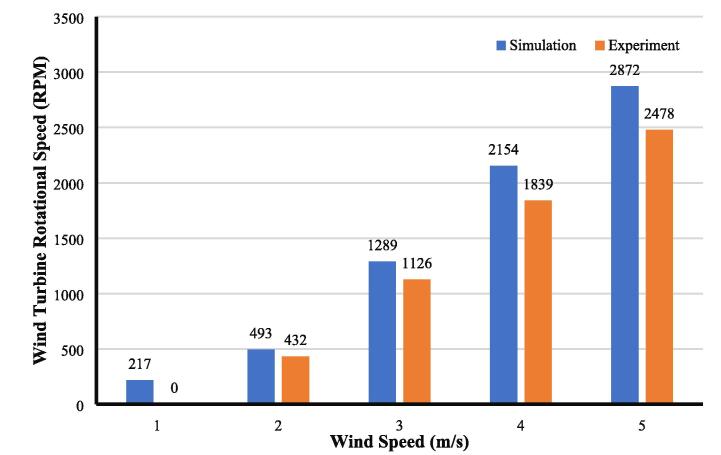
Results comparison from simulation and experiment for a single turbine.

Fig. 14 presents the rotor speed from actual measurement and software simulation. The rotor speed from actual measurement and software simulation varies, which can be considered normal for measurement in wind engineering. For instance, at a wind speed of 1 m/s, the rotor speed according to the simulation is 217 rpm, while the actual measurement shows no rotation from the rotor. During the actual measurement at a wind speed of 1 m/s, the rotor does not rotate as the inertia effect and torque stall is generally high, which is insufficient to spin the turbine. It only can be observed by using actual measurements inside the wind tunnel. It can also be seen considerably where the actual measurement always shows a smaller value than software simulation. It is a common phenomenon for wind measurement as the nature of wind is unpredictable and local turbulence likely occurs around the specimen. Nevertheless, the actual measurement indicates that the wind tunnel works appropriately and can be used to improve the quality of a single wind turbine design and compared by a software modeler to obtain a reliable design.

#### Inline configuration

**Fig. 15:**
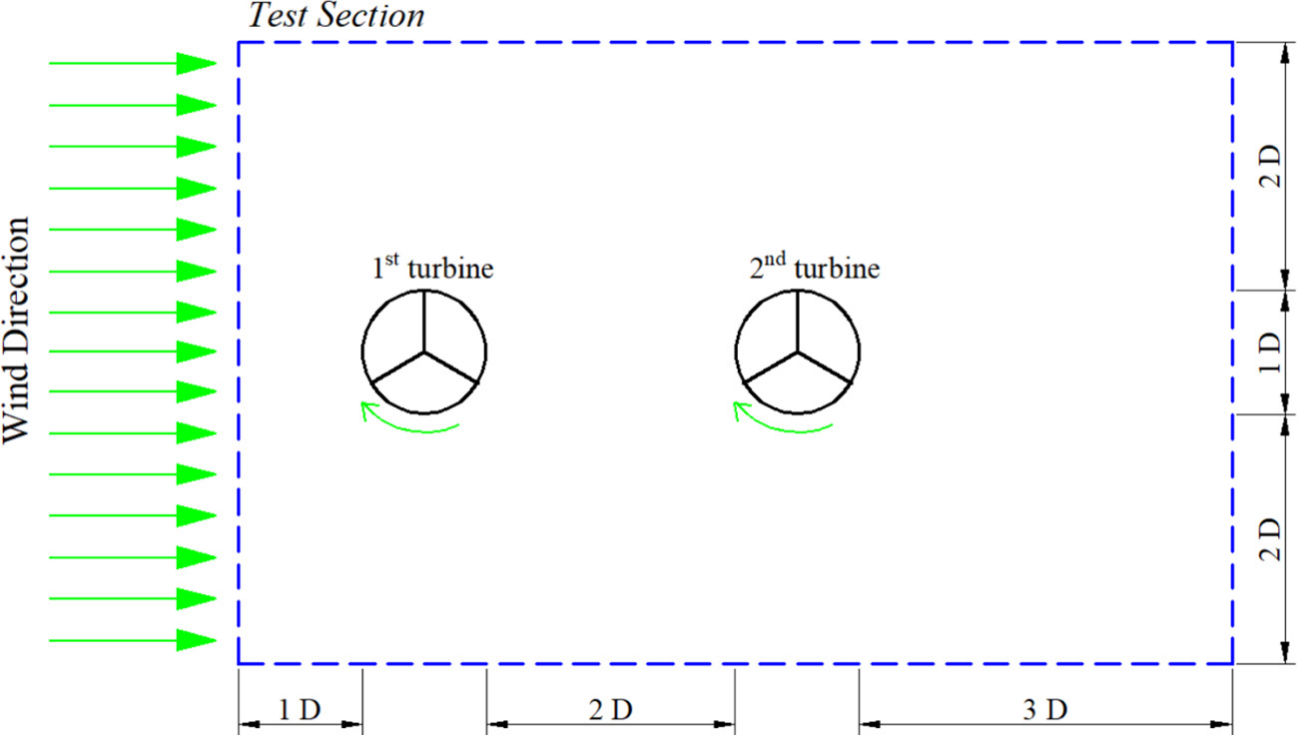
Rotor position for inline configuration.

The proposed wind tunnel has a spacious test section that can locate more than one specimen. It aims to accommodate further characterization for aerodynamic measurements, such as the effect of wake superposition for windfarm with more than one wind turbine. In this test, the measurement is intended for a wind turbine in inline configuration by locating the rotor, as seen in Fig. 15. The same rotor is placed in the test section and tested at a wind speed of 1–5 m/s. The simulation was also conducted similar to the specific parameter for the actual measurement.

**Fig. 16:**
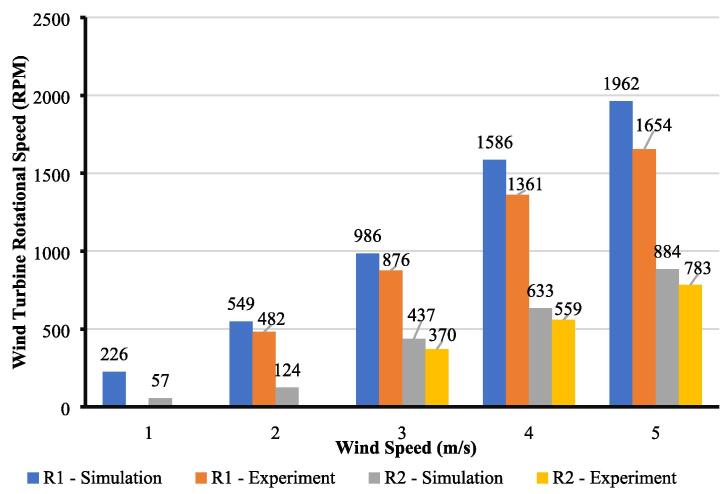
Results comparison between simulation and experiment for inline configuration.

Fig. 16 compares actual measurement and simulation for the rotor speed. First, it can be observed that the rotational speed of the first rotor (R_1_) is relatively higher at a wind speed of 1–2 m/s compared to the first characterization by using a single turbine (Fig. 14) and then decreases at a wind speed of 3–5 m/s. At low wind speed, the first rotor can absorb sufficient force from the wind and, since its position is closer to the entrance, can achieve a higher rotation speed. However, as the wind speed increases, the second rotor (R_2_) starts to rotate, causing an imbalance of wind distribution at the rear of R_1_ (wake superposition area), which decreases the rotational speed of the first rotor. Also, the deviation between actual measurement and simulation for R_1_ is relatively higher than a single turbine. On the contrary, the rotational speed of R_2_ increases at a higher wind speed with minimum deviation compared to simulation results. According to the result, the actual measurement can help the researcher better analyze by comparing the actual and simulation results. The study can help determine the optimal pitch distance for wind turbine configuration and can be further studied from this reference [22].

#### Staggered configuration

**Fig. 17:**
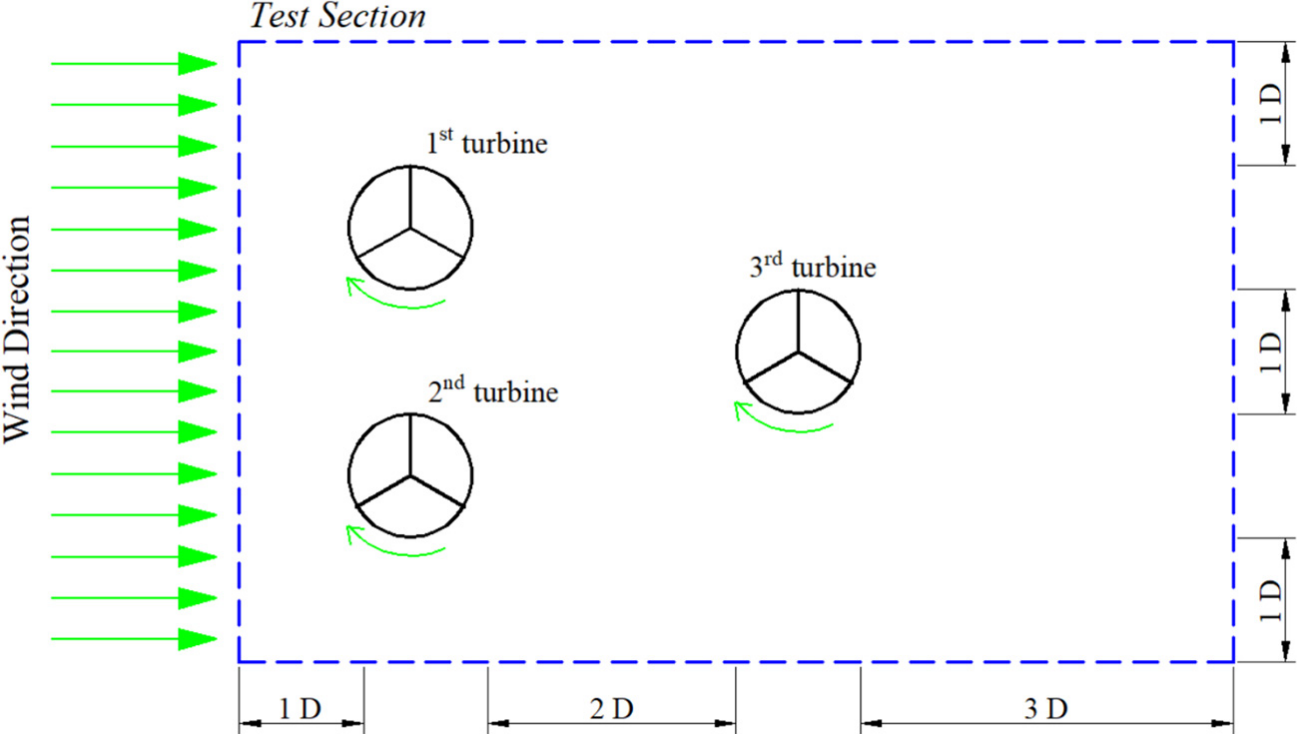
Rotor position for staggered configuration.

Perhaps, the most complicated arrangement for a wind turbine is a staggered configuration. It is the main reason why most of the studies focused on numerical simulation rather than actual measurement since staggered configuration requires a large area in the test section. With a vast space in the test section, it is possible to conduct an actual measurement for the staggered configuration. We also characterize the wind tunnel using a staggered configuration to validate the proposed design and provide a rigorous result. The layout for placing the wind turbine in the test section is shown in Fig. 17.

**Fig. 18:**
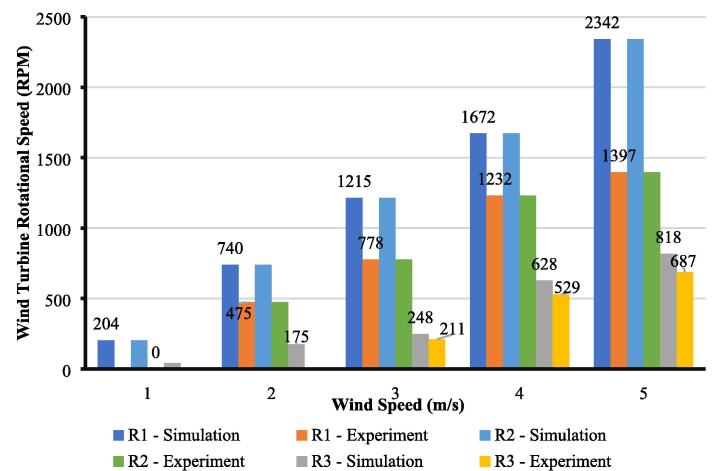
Results comparison between simulation and experiment for staggered configuration.

The result from the actual measurement and simulation is plotted in Fig. 18. The first-row turbine (R_1_ and R_2_) shows the same value in actual measurement and simulation. It indicates that each result shows a good agreement and can be said that the actual measurement is valid. The rotational speed shows the critical value from measurement for the second-row turbine (*R*_3_). For windspeed 1 and 2 m/s, the actual measurement indicates no rotational speed for the second-row turbine, while the simulation presents a low rotational speed. The actual measurement is relatively closed with the typical result for a staggered configuration where at low wind speed, the second-row turbine cannot produce sufficient rotational force due to the wake superposition effect. As the wind speed increases, the second-row turbine can rotate and the value from actual measurement and simulation varies relatively small. Thus, both results are comparable and can help analyze further while designing the layout for a wind farm. This reference can provide a detailed study of the effect of wake superposition and energy density for staggered configuration [23].

## Declaration of Competing Interest

The authors declare that they have no known competing financial interests or personal relationships that could have appeared to influence the work reported in this paper.
